# Endogenous control of fuelling in a migratory songbird

**DOI:** 10.1007/s00114-017-1514-0

**Published:** 2017-10-17

**Authors:** Ivan Maggini, Marc Bulte, Franz Bairlein

**Affiliations:** 1 Konrad-Lorenz Institute of Ethology, University of Veterinary Medicine, Vienna, Austria; 2 Institute of Avian Research “Vogelwarte Helgoland”, Wilhelmshaven, Germany

**Keywords:** Innate patterns, Inheritance, Fattening, *Oenanthe oenanthe*

## Abstract

The Northern wheatear (*Oenanthe oenanthe*) is a small long-distance migratory songbird that breeds throughout the Northern hemisphere and winters in sub-Saharan Africa. The main components of its migratory behaviour, i.e. seasonal migratory restlessness and body mass changes, have been shown to be under endogenous control. However, it is still unknown whether the disposition to accumulate fuel reserves is an inherited trait. We cross-bred Northern wheatears from two populations known to accumulate different amounts of fuel in a common-garden setup, and measured their maximum fuelling as the difference between the lightest and heaviest body mass recorded over each year for 4 years or longer. We used the largest value as a measure of maximum voluntary fuelling potential. F1-generation “hybrids” showed intermediate values to those of the parent populations. It was previously shown that in the wheatear the amount of fuel accumulated is closely linked to the presence of large ecological barriers to cross. This study shows that this adaptation has been fixed at the genetic level, and that intermediate traits are transferred to the next generation, with possible implications on the viability of such individuals in nature.

**Electronic supplementary material:**

The online version of this article (10.1007/s00114-017-1514-0) contains supplementary material, which is available to authorized users.

## Introduction

Migratory restlessness, the migration-related nocturnal activity of migratory songbirds in captivity (Gwinner 1968), has been shown to be genetically controlled and inherited (Berthold and Querner 1981). Species or populations of the same species with different migration distances show migratory restlessness which resembles the respective migration behaviour and migration distance in wild conspecifics (e.g. Berthold 1974; Berthold and Querner 1981; Gwinner 1990; Bulte and Bairlein 2013). It has also been shown that the total amount of fat accumulated by migrants prior to migration differs depending on the distance to be covered. In short/medium-distance migrants, fat accumulation is smaller compared to long-distance migrants which often double body mass prior to migration (Alerstam 1990; Battley et al. 2004; Gill et al. 2009). In contrast to migratory restlessness, however, migratory fat accumulation does not seem to directly reflect overall migration distance but rather the mode of migration and whether a species or population is confronted with an ecological obstacle such as sea or desert (Maggini and Bairlein 2010).

The Northern wheatear (*Oenanthe oenanthe*), hereafter wheatear, is a migratory songbird with a broad geographic breeding range, spanning large parts of the Holarctic. The *O. o. leucorhoa* subspecies breeds in Iceland, Greenland, and in eastern Canada, and the nominate *O*. *o*. *oenanthe* is found in Europe, Asia, and Alaska. The *O*. *o.* *seebohmi* subspecies is found in the Atlas Mountains of Northern Africa. All populations spend the winter in northern sub-Saharan Africa (Keith et al. 1996; Förschler et al. 2008), which means that some populations must cover enormous distances, as recently shown for wheatears from Alaska and Canada (Bairlein et al. 2012), whereas others fly much shorter distances. In addition, the birds from Canada, Greenland, and Iceland (*O. o.* *leucorhoa*) fly hundreds of kilometres across the northern Atlantic Ocean with no opportunities for resting and refuelling. Consequently, trans-oceanic wheatears have to store large amounts of fuel to be able to cross the barrier successfully (Bairlein and Gwinner 1994; Maggini and Bairlein 2010) while birds migrating over land regularly replenish fuel stores and keep lower fuel loads (Delingat et al. 2006).

The large variability in migratory phenotypes in the wheatear allows us to use this species for testing the genetic basis of fuelling in a common-garden experiment. Previous work showed that the diversity of patterns and extent of migratory behaviour is also revealed by wheatears in captivity (Bairlein et al. 2013). Captive naive wheatears from populations with different migratory routes and facing different ecological barriers showed endogenous population-specific differences in their migratory restlessness and other migratory traits such body mass variation (Maggini and Bairlein 2010) similar to what has been already reported for several other songbird species (Gwinner 1968; Berthold et al. 1972; Gwinner and Biebach 1977; Gwinner 1986; Holberton and Able 1992; Berthold 1996; Cadée et al. 1996; Helm and Gwinner 2006).

Two previous studies investigated the patterns of inheritance of body mass in a common-garden setup (Berthold and Querner 1982; Derégnaucourt et al. 2005). Both studies crossed birds from non-migratory with birds from migratory populations of the same species and found intermediate body mass patterns in the first generation of hybrids. In this study, we used long-term captive wheatears birds from two populations that both express migratory fattening but to a different extent (Maggini and Bairlein 2010) to confirm that fuelling traits are inherited and to test the hypothesis that first generation hybrids inherit intermediate traits. In contrast to previous studies, we did not focus on one single migration season but recorded yearly fuelling maxima by subtracting the minimum (lean) body mass from the maximum body mass reached by a single bird each year over 4+ years. We then took the largest of the measured maxima as a measure for maximum voluntary fuelling potential. This value shows the highest possible amount of fuel that is voluntarily accumulated by each individual, which we believe could be a measure of the physiological predisposition to accumulate (mostly) fat. In contrast to the previous studies, we did not focus only on the first year of life but on several years of life, in many cases throughout the whole life of an individual. To assess whether maximum fuelling is an inherited trait we cross-bred wheatears of two different populations with known differences in seasonal body mass patterns (Norway and Iceland; Maggini and Bairlein 2010). Migratory restlessness as well as other migratory traits has shown intermediate phenotypes during cross-breeding experiments (Berthold and Querner 1981; Helbig et al. 1994), so we predicted that the cross-bred offspring (hereafter F1-hybrids) would show intermediate maximum voluntary fuelling potentials with respect to those of their parent populations.

## Methods

We measured yearly minimum and maximum body mass, and the difference between the minimum and maximum body mass in birds from two populations of wheatears (Norway and Iceland) which were taken from their nests before fledging and then kept in a common-garden setup for their entire life. We measured the same variables in birds from cross-broods between the two populations and in pure Norwegian offspring. The maximum difference between minimum and maximum yearly body mass was used as a measure of maximum voluntary fuelling potential. For details on the methods see the Electronic Supplementary Materials.

## Results and discussion

There was a moderate year-to-year repeatability in the measurement of minimum body mass (63% [CI95, 50–75%]), maximum body mass (74% [63–83%]), and in the difference between minimum and maximum body mass (49% [35–64%]).

**Fig. 1:**
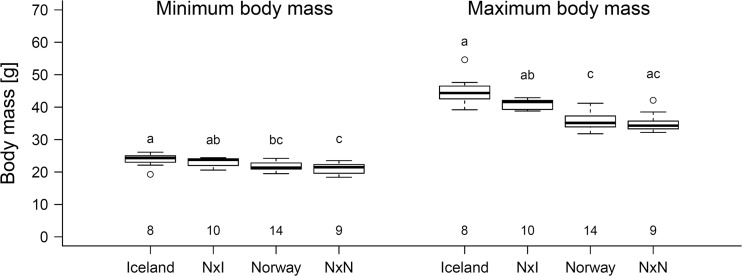
Minimum and maximum body mass in the four groups studied. Icelandic and Norwegian birds were born in the wild, while Norway x Iceland and Norway x Norway birds were from the F1 generation bred in captivity. Sample sizes are given below the boxplots. Letters above the boxplots indicate significant differences among groups. Not that for maximum body mass, the statistical tests refer to differences in maximum voluntary fuelling potential and not to the actual maximum mass values (i.e. they account for initial body mass). Minimum body mass was 23.85 ± 0.76 g for Icelandic birds, 23.10 ± 0.41 g for Norway x Iceland offspring, 21.69 ± 0.36 g for Norwegian birds, and 21.03 ± 0.56 g for pure Norwegian offspring. Maximum voluntary fuelling potential was 21.33 ± 1.50 g for Icelandic birds, 18.00 ± 0.22 g for Norway x Iceland offspring, 14.28 ± 0.63 g for Norwegian birds, and 14.37 ± 0.92 g for pure Norwegian offspring

There were significant differences among populations and F1 generations in minimum body mass (LMM: F_3,15_ = 6.64, *p* = 0.005, Fig. 1, Table S1). Furthermore, males had higher minimum body mass than females (LMM: F_1,21_ = 7.59, *p* = 0.012). Post hoc tests revealed significant differences between the two parent populations (Iceland vs. Norway, *p* = 0.003), between the F1 pure Norwegian offspring and the Icelandic birds (*p* < 0.001), and between the F1-hybrids and the F1 pure Norwegian offspring (*p* = 0.015). No differences were found between the F1-hybrids and their parental populations (Norway x Iceland vs. Iceland, *p* = 0.407; Norway x Iceland vs. Norway, *p* = 0.213), nor between the Norwegian parents and the F1 pure Norwegian offspring (*p* = 0.554). This result highlights differences in minimum body mass between populations, most likely as a result of differences in structural size. The way these differences are transmitted to the next generation is unclear. The F1-hybrids had values closer to those of their Icelandic parents, which might indicate possible maternal effects, since only one of the ten hybrids was born from an Icelandic father.

There was a significant difference in maximum voluntary fuelling potential among populations and F1 generations (LMM: F_3,15_ = 12.11, *p* < 0.001, Fig. 1, Table S2), but no difference between sexes (LMM: F_1,20_ = 0.89, *p* = 0.357). The differences were significant between the two parental populations (Iceland vs. Norway, *p* < 0.001), between the F1 pure Norwegian offspring and the Icelandic birds (*p* < 0.001), and between the F1-hybrids and the Norwegian population (*p* = 0.024). The difference between the F1 pure Norwegian offspring and the Icelandic birds was marginally non-significant (*p* = 0.075), as was the difference between the F1-hybrids and the Icelandic population (*p* = 0.067). There was no significant difference between the Norwegian parents and their pure F1 offspring (*p* > 0.5). Minimum body mass had no significant effect on maximum voluntary fuelling potential (LMM: F_1,20_ = 0.14, *p* = 0.715), indicating that maximum voluntary fuelling potential does not depend on structural mass, and is developed as a response to specific population needs. This is in line with previous results (Maggini and Bairlein 2010; Bulte and Bairlein 2013) that showed that the high fuel loads carried by Icelandic birds relate to their need to cross the open sea during their migration. The comparison between expected and observed values of maximum voluntary fuelling potential of F1 offspring showed no significant deviation from the expected intermediate values (one-sample *t* test, *t* = − 0.55; df = 29.9; *p* = 0.59). This confirms the prediction that among population differences in maximum voluntary, fuelling potential are inherited directly. Although we cannot completely exclude that early environmental effects might play a role, this seems unlikely. The birds which were taken from the wild in both Norway and Iceland were taken from their nests at 5–9 days of age. However, the pure Norwegian F1 offspring that were born in the common-garden setup did not differ from their parents born in the wild. This suggests no such effect. A difference in early investment by the parents also seems to be an unlikely explanation for the observed intermediate values. If the parents of hybrids would invest more in their offspring, then the offspring would be expected to be more similar to the most influential parent, in our case probably the Icelandic parent, since they are the ones with higher fuelling. It is unlikely that early parental investment could play a role in the potential to refuel later in life, especially since this can be expressed even several years after birth. However, this possibility could be investigated by performing cross-fostering experiments.

With this study, we show that fuel storing in a long-distance migratory songbird is inherited. F1-hybrids of parental populations with significantly different migratory fuelling capacities exhibited intermediate values of fuelling capacity as compared to their respective parent populations. Being an inherited trait, migratory fuelling potential is exposed to selection and thus worth being further considered in the light of recent global climate change (Walther et al. 2002). The genetic basis of physiological traits has important implications on adaptability and micro-evolutionary processes in migratory birds (Bairlein and Hüppop 2004).

## Electronic supplementary material

ESM 1 (DOCX 17 kb)
