# Supplementary material for: Endogenous control of fuelling in a migratory songbird
Source: Naturwissenschaften. 2017 Oct 17;104(11):93. doi: 10.1007/s00114-017-1514-0 (PMC5644693; doi:10.1007/s00114-017-1514-0)
Supplement: Supplementary file 1 — (DOCX 17 kb) [file 114_2017_1514_MOESM1_ESM.docx]

**Electronic Supporting Materials to:**

**Endogenous control of fuelling in a migratory songbird**

by

Ivan Maggini, Marc Bulte, and Franz Bairlein

Methods

The parental wheatears used for this study were taken under licence from their nests in the breeding areas. Icelandic birds were taken in the Myvatn Lake area (65°39’N, 16°36’W) on 29 June 2005 and in Öxnadalðalur (65°37’N, 18°29’W) between 3 and 7 July 2006. Norwegian birds were taken in Rogaland (58°43’N, 6°12’E) on 26 and 27 June 2005 and in Norddalen (60°51’N, 6°11’E) on 3 July 2006. They were taken from the nest as 6-9 days old nestlings, then transported within 1-2 days to the Institute for Avian Research in Wilhelmshaven (Germany) and kept indoors in individual cages (size 50x40x40cm). The tested hybrids were F1 offspring of the abovementioned birds. These were bred in outdoor aviaries (one pair per aviary of circa 4x3x3m) in the natural breeding period on the grounds of the Institute for Avian Research and taken indoors as soon as they were independent. Birds from the original populations as well as the F1-hybrids were kept in individual cages, except for several weeks in spring and summer when the birds from the original populations were transferred to outdoor aviaries to breed. From the autumn migration period until early spring all birds were kept indoors under controlled conditions (light cycle 12 h light:12 h dark; temperature 20±1 °C). This is the time when migratory fuelling occurs (Maggini and Bairlein 2010). A standardized insectivore diet (Bairlein 1986) supplemented with a few mealworms, and freshwater were provided ad libitum. Body mass was recorded twice a week to the nearest 0.1 g, early in the morning before the birds were offered food. This was done over the entire lifetime of the birds, except for the periods the birds spent in the aviaries breeding.

Every year we calculated the difference between minimum (as a proxy for lean mass) and maximum body mass. We voluntarily did not put constraints on when during the year these two values were obtained, since we were interested in the absolute maximum difference between them. Mostly, the lowest body mass was recorded during postbreeding moult, and the highest during autumn migration (pers. obs.), but this was not always the case. We then considered the highest yearly value of each individual as the maximum voluntary fuelling potential. This was done for two different populations (Iceland and Norway) and for their F1-hybrids (Norway x Iceland). To exclude the effect of captive breeding on the results of the F1-hybrids we also bred a group of Norway x Norway to serve as a control. Only birds for which we had at least four years of data were included in the analysis. We chose the four-year threshold because this was the minimum number of years that we had from our captive birds, if we excluded birds with only one or two years of data (i.e. we had no birds with three years of data). Repeatability was rather low when birds were measured for two years only, therefore we removed them from the sample. In total, we obtained measurements from 8 birds from Iceland (3 born in 2005, 5 in 2006), 14 from Norway (5 born in 2005, 9 in 2006), 10 Norway x Iceland hybrids (4 born in 2007, 6 in 2008), and 9 Norway x Norway offspring (1 born in 2007, 8 in 2009). We calculated intra-individual repeatability of the measurement of lean and maximum body mass, and on the difference between minimum and maximum body mass using Intraclass Correlation Coefficients (ICC) from the ICC package in R (Wolak et al. 2012). We tested for differences between populations in minimum body mass and maximum voluntary fuelling potential using linear mixed effects models with population and sex as fixed factors and nest as random factor. In the model for maximum voluntary fuelling potential, we also added minimum body mass as a covariate. Pairwise comparisons with Tukey corrections were then performed on the full models to identify significant differences between groups, using the glht function in the multcomp package in R (Hothorn et al. 2008). We also tested whether the F1 generation would have intermediate values to those of their parents using a one-sample t-test comparing the actual maximum voluntary fuelling potential of the offspring with the expected value that was obtained from the average of the values for both parents. All analyses were performed using R 3.3.2 (R Core Team 2016).

All animals were handled according to international ethical standards. Licences for capture were granted from the Ministry for the Environment (Iceland, licence nr. UMH05030044/13-4-1 HS/– and UMH06040104/13-4-1 of 12 May 2006) and the Directorate for Nature Management (Norway, licence nr. 05/ 3039 ART-VI-ARES of 28 April 2005 and 2005/3039 ART-VIARES of 3 May 2006). The Institute of Avian Research has a general permission for housing birds from the Landesregierung Niedersachsen (LAVES: 509f-42502-32/12 of 30 July 2004), and no additional permissions are required in Germany for behavioural studies.

Results

Tab. S1. Parameter estimates for the LMM of lean body mass. Random effect nest: SD <0.001, residual 1.47.

| Variable | Estimate | SE | DF | t-value | p-value |
| --- | --- | --- | --- | --- | --- |
| **Population:** |  |  |  |  |  |
| Iceland (intercept) | 23.42 | 0.53 | 21 | 44.06 | <0.001 |
| Norway | -2.29 | 0.66 | 15 | -3.50 | 0.003 |
| Nor x Icel | -1.11 | 0.71 | 15 | -1.55 | 0.142 |
| Nor x Nor | -3.12 | 0.73 | 15 | -4.29 | <0.001 |
| **Sex** | 1.31 | 0.47 | 21 | 2.75 | 0.012 |

Tab. S2. Parameter estimates for the LMM of maximum voluntary fuelling potential. Random effect nest: SD = 0.96, residual 2.57.

| Variable | Estimate | SE | DF | t-value | p-value |
| --- | --- | --- | --- | --- | --- |
| **Population:** |  |  |  |  |  |
| Iceland (intercept) | 23.59 | 7.10 | 20 | 3.32 | 0.003 |
| Norway | -7.37 | 1.46 | 15 | -5.04 | <0.001 |
| Nor x Icel | -3.62 | 1.48 | 15 | -2.44 | 0.027 |
| Nor x Nor | -7.42 | 1.75 | 15 | -4.24 | <0.001 |
| **Sex** | 0.96 | 0.95 | 20 | 1.01 | 0.324 |
| **Lean mass** | -0.11 | 0.30 | 20 | -0.37 | 0.715 |

References

Bairlein F (1986) Ein standardisiertes Futter für Ernährungsuntersuchungen an omnivoren Kleinvögeln. J Ornithol 127:338-340.

Hothorn T, Bretz F,Westfall P (2008). Simultaneous inference in general parametric models. Biometr J 50:346-363.

Maggini I, Bairlein F (2010) Endogenous rhythms of seasonal migratory body mass change and nocturnal restlessness in different populations of Northern Wheatear *Oenanthe oenanthe*. J Biol Rhythms 25:268-276.

R Core Team (2016) R: A language and environment for statistical computing. R Foundation for Statistical Computing, Vienna, Austria. URL <https://www.R-project.org/>.

Wolak ME, Fairbairn DJ, Paulsen YR (2012) Guidelines for estimating repeatability. Meth Ecol Evol 3:129-137.
